# Association between patient position-induced breast shape changes on prone and supine MRI and mammographic breast density or thickness

**DOI:** 10.1007/s11604-024-01708-y

**Published:** 2024-11-25

**Authors:** Maki Amano, Yasuo Amano, Naoya Ishibashi, Takeshi Yamaguchi, Mitsuhiro Watanabe

**Affiliations:** 1 Department of Radiology, Nihon University Hospital, Tokyo, Japan; 2 College of Science and Technology, Nihon University, Chiba, Japan

**Keywords:** Breast MRI, Mammography, Breast shape change, Mammographic breast density, Compressed breast thickness

## Abstract

**Purpose:**

The breast shape differs between the prone position in breast magnetic resonance imaging (MRI) and the supine position on an operating table. We sought to determine the relationship between patient position-induced changes on prone and supine MRI in breast shape and mammographic breast density or thickness.

**Materials and methods:**

We evaluated data from 68 women with 69 breast cancers in this retrospective observational study. The difference in the minimal distance from the nipple to the pectoralis major (DNPp-s) or the internal thoracic artery between the prone and supine MRI (DNIs-p) was defined as the breast shape changes. Mammographic breast density was assessed by conventional 4-level classification and automated and manual quantification using a dedicated mammography viewer. The compressed breast thickness was recorded during mammography (MMG). We determined the association between patient position-induced breast shape changes on MRI and mammographic breast density or compressed breast thickness on MMG.

**Results:**

On the conventional 4-level qualification, one breast appeared fatty, 39 appeared with scattered density, 23 appeared heterogeneously dense, and 6 breasts appeared extremely dense. Both automated and manual quantification of mammographic breast density differed between the 4 levels (*p* < 0.01 for both) and correlated with the 4 levels (*p* < 0.001 for both,* r* = 0.654 and 0.693, respectively). The manual quantification inversely correlated with DNPp-s and DNIs-p (*p* < 0.01 and < 0.05, *r* =  − 0.330 and − 0.273, respectively). The compressed breast thickness significantly correlated with DNPp-s and DNIs-p (*p* < 0.01 for both, *r* = 0.648 and 0.467, respectively).

**Conclusion:**

Compressed breast thickness during MMG can predict the degree of patient position-induced changes in breast shape on MRI. The manual quantification of the mammographic breast density, which may reflect the biomechanical properties of the breast tissues, also correlates to the breast shape changes.

## Introduction

Magnetic resonance imaging (MRI) is the best imaging method to evaluate the spread of breast cancer in planning breast-conserving surgery because of its high spatial and contrast resolution [1, 2]. Breast MRI is routinely performed with the patient prone to stretch the whole breast fully and to ensure a high image quality. However, the breast shape when the patient is prone during MRI differs from that when they are supine on an operating table. The location and extent of the breast cancer seen on MRI may not necessarily coincide with those during the surgery, which may be an important limitation of preoperative MRI.

There are several techniques to display an MRI of the breast during surgery, including real-time virtual sonography [3, 4] and MRI projection mapping [5]. These techniques require an additional contrast-enhanced supine MRI. Simulation techniques that generate supine MRI from prone MRI might avoid an additional supine MRI examination [6–9]. However, breast shape changes between the two acquisition positions can vary widely between the patients and should be addressed for precise simulation. The biomechanical properties of breasts, including tissue softness and size, may be related to the degree of breast shape changes associated with a change in patient position from prone to supine.

Mammography (MMG) is essential for screening and diagnosing breast cancer. MMG shows that the breast is primarily composed of fibroglandular tissue and fat. MRI elastography reveals that the fat is softer than the fibroglandular tissue [10, 11], and the proportion of the two tissue components, that is, the mammographic breast density, may reflect the breast softness. Dedicated MMG viewers can quantify the mammographic breast density successfully. They quantitatively depict dense breasts with abundant fibroglandular tissue, which is known as a risk factor for breast cancer [12, 13]. In clinical practice, the distance between the detector and the compression plate during MMG is recorded as the breast thickness, which is generally considered a parameter reflecting the breast size.

We hypothesized that the mammographic breast density and compressed breast thickness on MMG are related to the degree of breast shape changes when the patient’s position shifts from prone to supine on MRI. Thus, we sought to assess the association between the patient position-induced breast shape changes on MRI and the mammographic breast density or compressed breast thickness on MMG.

## Materials and methods

The present retrospective observational study was approved by our Institutional Review Board (No. 202200403). Any requirement for informed consent from the patients was specifically waived because of the retrospective nature of this study.

### Study population

We recruited 88 women with 89 breast cancers who underwent MMG and MRI from April 2021 to June 2024; breast MRI studies comprised breast MRI for routine diagnosis when the patients were prone and breast MRI when the patients were supine for MRI projection mapping to support breast-conserving surgery [5]. All of them underwent the surgery one day after the supine MRI. The exclusion criteria were as follows: (1) using a tilting table (10°) on supine MRI that prevented measuring the breast shape changes in the mediolateral direction, (2) breast cancers involving the skin or pectoralis muscle or that of T3 (i.e., ≥ 5 cm), which might affect the degree of the breast shape changes, and (3) low image quality of MMG or MRI.

### Imaging procedures

The interval between MMG and prone MRI ranged from 3 to 221 days (median 18 days; IQR 14–27 days), while the interval between prone and supine MRI ranged from 1 to 245 days (median 22 days; IQR 14–36 days).

Fifty-two MMG examinations including 53 affected breasts were performed using a Pe.ru.ru (Canon Medical Systems, Tokyo, Japan) in our hospital. The remaining 16 examinations were performed in other institutions. Mediolateral and craniocaudal views were acquired under the compression by radiological technologists.

Sixty-five prone breast MRI examinations were performed using a 3.0 T system (Ingenia; Philips Healthcare, Best, The Netherlands) in our hospital. The other 3 MRIs were taken in the other institutions (3.0 T, 2; 1.5 T, 1). The prone breast MRI was performed using a standard clinical MRI protocol to make a clinical diagnosis of breast cancers or delineate the location and extent of the tumors. The prone breast MRI study consisted of pre- and post-contrast T1-weighted imaging in the transverse, coronal and sagittal planes, and transverse T2-weighted and diffusion-weighted imaging sequences. The pre-contrast T1-weighted images in the prone position were acquired using standard two-dimensional turbo spin-echo imaging owing to its high image quality, while the post-contrast T1-weighted images were obtained with the dynamic enhanced fat-suppressed three-dimensional (3D) gradient-echo imaging. In all of the patients, the supine MRI was performed in our institution one day before the breast-conserving surgery to determine the location and extent of the breast cancer in a position identical to that used in the operating room [5]. MRI in the supine position covered the unilateral breast with breast cancer involvement, and the patient’s arms were raised using an in-house custom-made armrest, which allowed the position of the arms to be the same as that used in the operating room. Pre- and post-contrast T1-weighted images in the supine position were performed using breath-hold 3D gradient-echo imaging with identical spatial resolution. Table 1 summarized the receiver coils used, T1-weighted imaging sequences, and their typical imaging parameters used in the prone and supine MRI.

**Table 1:** Receiver coil and T1-weighted imaging parameters for the prone and supine breast MRI

Position	Coil	Breath hold	CE	T1WI	Fat suppression	TR/TE (ms)	FA	FOV (mm)	Matrix	Thickness (mm)	Scan time (s)
Prone	Breast 16 ch	−	pre	TSE	−	562/10	90	340	512	4	61
			post	GRE	+	3.9/2.0	11	340	560	2	60
Supine	Torso 32 ch	+	pre	GRE	−	3.5/1.7	10	340	704	3	18
			post		+	3.5/1.7	10	340	704	3	20

CE, contrast enhancement; T1WI, T1-weighted imaging; TR, repetition time; TE, echo time; FA, flip angle; FOV, field of view; ch, channel; pre, pre-contrast; post; post-contrast; TSE, turbo spin-echo; GRE, gradient-echo. Fat suppression was used in contrast-enhanced three-dimensional GRE

### MMG analysis

**Fig. 1:**
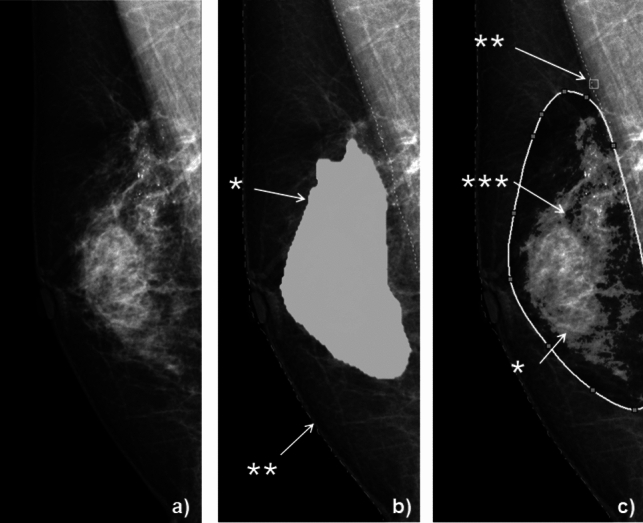
Estimation of mammographic breast density. **a** Scattered density on the conventional 4-level classification. **b** Automated quantification is calculated as the breast tissue* identified automatically divided by the whole breast**. Automated quantification is 25.7% in this patient. **c** For manual quantification, the denominator is defined as the area where the original breast tissue is thought to exist (drawn contour). The fibroglandular tissue is delineated as the region* with a density equal to or higher than the pectoralis major muscle by placing a point** on the muscle, and the fat*** intervening fibroglandular tissue is excluded from the region when calculating the numerator. The automated quantification is 46.3% in this patient

The mammographic breast density on the mediolateral view was estimated using a dedicated MMG viewer system (mammodite^®^, NetCam, Tokyo, Japan) with one visual classification and two quantification methods (Fig. 1) as follows:

1. Conventional 4-level classification recommended by the Japan Central Organization on Quality Assurance of Breast Cancer Screening (QABCS, https://www.qabcs.or.jp/news/20200206.html) was used to evaluate the breast density on MMG. In this classification, the denominator is defined as the area where the original breast tissue is thought to exist while excluding the subcutaneous and retromammary fat and the pectoralis major muscle, and the numerator is visually defined as the sum of the areas with density identical to or higher than the pectoralis major muscle in the denominator noted above. The ratio of the numerator to the denominator was assessed visually and classified into the following 4 levels: fatty, the ratio < 10%; scattered density, 10–50%; heterogeneous dense, 50–80%; and extremely dense, ≥ 80%.
2. Automated quantification (%) of the breast density was obtained using standard software installed in mammodite^®^. The denominator was the area of the whole breast, and the numerator was the breast tissue identified automatically. The numerator of the automated quantification could include the fat intervening within the fibroglandular tissue.
3. Manual quantification (%) of the breast density was obtained using the other software installed in mammodite^®^ and manual correction. First, according to the conventional 4-level classification, the area of the original breast tissue was manually drawn as the denominator. Next, the fibroglandular tissue of the breast was delineated as the region with a density equal to or higher than the pectoralis major muscle by placing a point within the muscle, showing the representative density as the muscle; the fat intervening the fibroglandular tissue was excluded from the numerator in the manual quantification.

A board-certified radiologist, who had 13 years’ experience in breast imaging and a class A certification awarded by the QABCS, evaluated breast density. The compressed breast thickness was recorded during MMG.

### MRI analysis

**Fig. 2:**
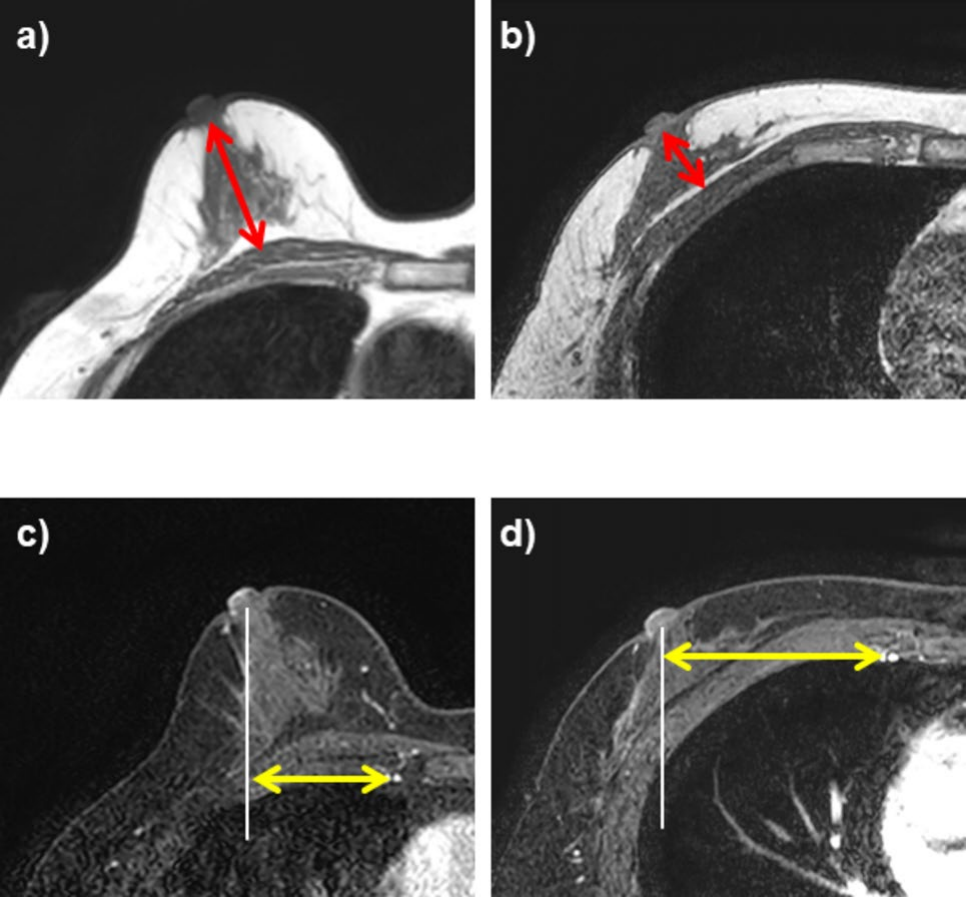
The minimal distance from the nipple to the pectoralis major both in the prone (**a**) and supine MRI (**b**). The degree of breast shape changes in the anterior–posterior direction is defined as the difference in the minimal distances between the two positions. The distance from the nipple to the internal thoracic artery in the prone (**c**) and supine breast MRI (**d**). The distance is measured as that between the vertical line from the center of the nipple base (white line) and the internal thoracic artery (yellow arrow). The degree of breast shape changes in the lateral direction is defined as the difference in the distance from the nipple to the internal thoracic artery between the two positions

The same radiologist and the other with 30 year-experience in diagnostic MRI estimated the breast shape changes in anterior–posterior direction of the breast and the mediolateral direction of the chest on the breast MRI while the patient was prone and supine. First, the two independent radiologists measured the minimum distances from the nipple to the pectoralis major muscle (DNP, in mm) when the patient was prone (DNPp, in mm) and supine (DNPs, in mm) on the transverse T1-weighted MR images without fat suppression using a Picture Achieving and Communication System (Centricity, GE Healthcare, Milwaukee, WI, USA) (Fig. 2a, b). Two-dimensional turbo spin-echo imaging was used in the prone MRI, while three-dimensional gradient-echo imaging was used in the supine MRI. The degree of breast shape changes in the anterior–posterior direction (i.e., DNPp-s, in mm) was defined as the difference between DNPp and DNPs. Next, the degree of the breast change shape in the mediolateral direction was defined as the change in the distance from the nipple to the internal thoracic artery between the prone and supine breast MRI (DNIs-p, in mm; Fig. 2c, d). In brief, a horizontal line passing the internal thoracic artery and a vertical line from the center of the nipple base were drawn on the transverse fat-suppressed contrast-enhanced MRI. Then, the distance between the intersection of the lines above and the internal thoracic artery was measured and defined as the distance from the nipple to the internal thoracic artery. The distances from the nipple to the internal thoracic artery (DNI) were measured on the prone and supine MRI (DNIp and DNIs, respectively), and DNIs-p was calculated as DNIs minus DNIp.

### Statistical analysis

We checked the distribution of the numerical data using Kolmogorov–Smirnov test; mean and SD were shown for parametric data, while median and interquartile range (IQR) were shown for non-parametric data. We first assessed the differences in the mammographic breast density measured by the automated or manual quantification between the conventional 4-level classification using a Kruskal–Wallis test with a post hoc Bonferroni correction because the visual classification had been established by QABCS. We also assessed the correlation of the mammographic breast density between the conventional 4-level classification and the automated or manual quantification. Thereafter, we assessed the correlation between the breast shape changes on breast MRI, including DNPp-s and DNIs-p, and the mammographic breast density measured by the automated or manual quantification or the compressed breast thickness on MMG. Correlations were analyzed using Pearson or Spearman’s rank test when appropriate. All statistical analyses were performed using IBM SPSS Statistics for Windows (version 26; IBM Corp., Armonk, NY, USA). A *p* < 0.05 was considered significant.

## Results

### Patients eligible for imaging analysis

We excluded 20 patients from further study because the supine MRI was done using a tilting table (*n* = 8) [5], breast cancers involving the skin or pectoralis muscle or that of T3 (*n* = 6), or low image quality of MMG or MRI (*n* = 4). In addition, we excluded 2 patients who underwent treatments before supine MRI.

Consequently, data from a total of 68 women with 69 breast cancers were analyzed. The patients’ ages ranged from 31 to 77 years, with a median age of 51 years [interquartile range (IQR) 44–62.5 years]. The pathohistological types of the 69 breast cancers determined by vacuum assisted biopsy (*n* = 43) or core needle biopsy (*n* = 26) were as follows: invasive ductal carcinomas (IDC), 48; ductal carcinomas in situ (DCIS), 14; IDC with predominant DCIS, 2; invasive lobular carcinoma, 3; mucinous carcinoma, 1; and myoepithelial carcinoma, 1. Immunohistopathological subtypes of 55 invasive breast cancers were as follows: luminal A, 17; luminal B, 18; HER2, 13; triple negative, 7. The clinical stagings of 69 cancers were as follows: cTis, 14; cT1aN0, 2; cT1bN0, 5; cT1cN0, 21; cT1cN1, 1; cT2N0, 13; cT2N1,10; cT2N2,2 and cT2N3, 1. None had distant metastasis. The 69 cancers are located in the breast as follows: inner-upper,16; inner-medial,4; inner-lower, 3; outer-upper, 31; outer-medial, 5; outer-lower, 5; upper-medial, 3; lower-medial, 2.

### Quantification of mammographic breast density

**Fig. 3:**
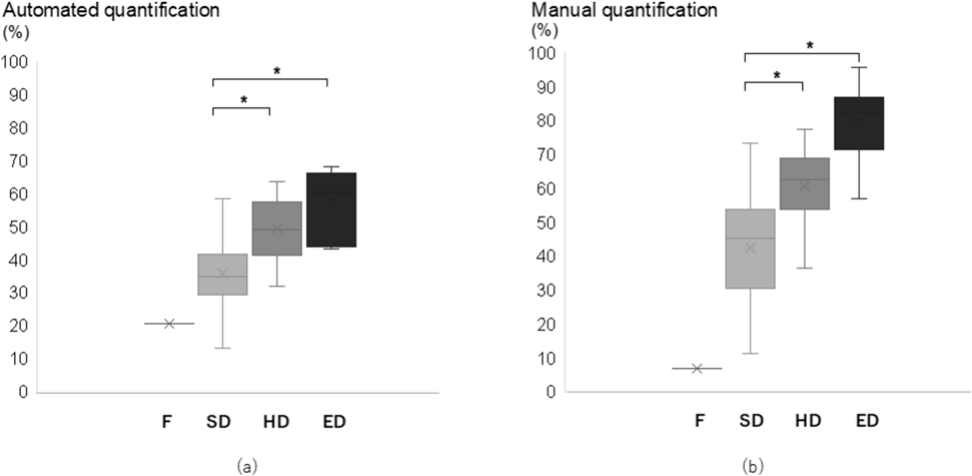
F, fatty; SD, scattered density; HD, heterogeneously dense; ED, Extremely dense. There are significant differences in the automated (**a**) or manual quantification (**b**) between the 4-level classification (*p* < 0.01). SD group shows significantly lower mammographic breast density than HD and ED groups in both the automated (**a**) and manual quantification (**b**) (*p* < 0.01 for both)

The conventional 4-level classification was distributed as follows: fatty, 1 (1.4%); scattered density, 39 (56.5%); heterogeneously dense, 23 (33.3%); and extremely dense, 6 (8.7%). Automated quantification ranged from 13.0 to 68.1%, with a mean of 41.8%, and manual quantification ranged from 7.0 to 95.6%, with a mean of 51.4%. We found significant differences in the automated or manual quantification between the 4-level classification (*p* < 0.01). A post hoc correction revealed that the scattered density group showed significantly lower mammographic breast density than the heterogeneously and extremely dense groups in both the automated and manual quantification (*p* < 0.01 for both, Fig. 3a, b). Significant correlations were observed between the 4-level classification and the automated or manual quantification (*r* = 0.654 and 0.693, respectively, *p* < 0.01 for both).

### Association between the breast shape changes and mammographic breast density or compressed breast thickness

**Fig. 4:**
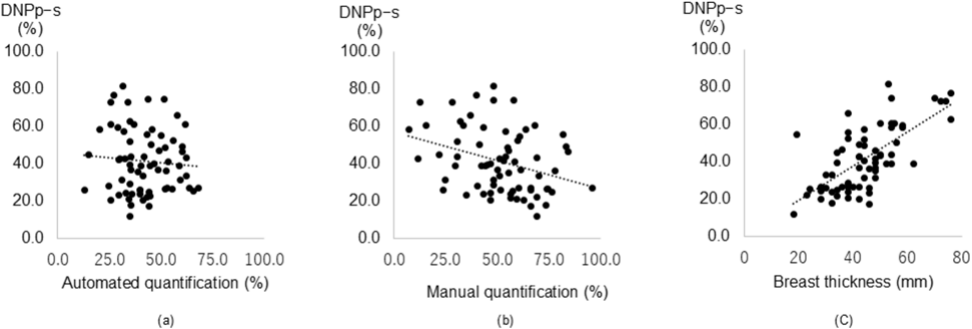
DNPp-s reflects the breast shape changes in the anterior–posterior direction. Although the automated quantification of mammographic breast density does not correlate with DNPp-s (**a**, *p* = 0.716), the manual quantification (**b**) and compressed breast thickness (**c**) correlate with DNPp-s (*r* =  − 0.330 and *p* < 0.01 for manual quantification and *r* = 0.648 and *p* < 0.01 for compressed breast thickness)

**Fig. 5:**
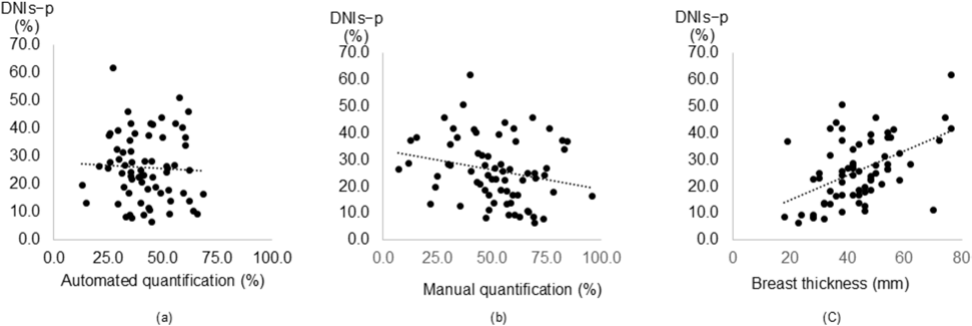
DNIs-p reflects the breast shape changes in the mediolateral direction. The automated quantification of mammographic breast density does not correlate with DNIs-p (**a**, *p* = 0.670), whereas the manual quantification (**b**) and compressed breast thickness (**c**) correlate with DNIs-p (*r* =  − 0.273 and *p* < 0.05 for manual quantification and *r* = 0.467 and *p* < 0.01 for compressed breast thickness)

The interclass correlation was high for DNPp, DNPs, DNPp-s, DNIp, DNIs and DNIs-p (*r* = 0.995, 0.968, 0.985, 0.939, 0.987 and 0.974, respectively) between the two independent radiologists (*p* < 0.01 for all). Because these indicated strong consistency, the average values are shown in Table 2. The average values of DNPp-s and DNIs-p were used for the following analyses. Table 2 also showed the automated and manual quantification of mammographic breast density and compressed breast thickness on MMG. Table 3 summarizes the correlations between the patient position-induced breast shape changes and the quantified mammographic breast density or the compressed breast thickness. The DNPp-s indicating the anterior–posterior change of the breast shape inversely correlated with the manual quantification of the mammographic breast density (*r* =  − 0.330, *p* < 0.01) and positively correlated with the compressed breast thickness (*r* = 0.648, *p* < 0.01) but not with the automated quantification of the mammographic breast density (Fig. 4). The DNIs-p indicating the lateral change of the breast shape inversely correlated with the manual quantification of the mammographic breast density (*r* =  − 0.273, *p* < 0.05) and positively correlated with the compressed breast thickness (*r* = 0.467, *p* < 0.01) but not with the automated quantification of the mammographic breast density (Fig. 5).

**Table 2:** Summary of MRI and MMG analyses

	Range	Mean ± SD	Median (IQR)
DNPp (mm)	27.5–134		63 (47.8–80.8)
DNPs (mm)	13–61		25.5 (20.8–33.0)
DNPp-s (mm)	12–82		39 (26.3–53.8)
DNIp (mm)	36–82	54.8 ± 8.5	
DNIs (mm)	56–117.5	80.8 ± 13.6	
DNIs-p (mm)	6.5–62	26.0 ± 12.0	
Mammographic breast density (%)			
Automated quantification	13.0–68.1	41.9 ± 12.6	
Manual quantification	7.0–95.6	51.4 ± 18.6	
Compressed breast thickness (mm)	18–76	43.9 ± 12.8	

MRI, magnetic resonance imaging; MMG, mammography; SD, standard deviation; IQR, interquartile range; DNPp, the minimal distance from the nipple to the pectoralis major muscle in prone position; DNPs, the minimal distance from the nipple to the pectoralis major muscle in supine position; DNPp-s, DNPp minus DNPs; DNIp, distance from the nipple to the internal thoracic artery in prone position; DNIs, distance from the nipple to the internal thoracic artery in supine position; DNIs-p, DNIs minus DNIp

**Table 3:** Correlation between the breast shape changes and the quantified mammographic breast density or the compressed breast thickness

Correlation with breast shape changes	DNPp-s (mm)		DNIs-p (mm)	
	*r*	*p*	*r*	*p*
Mammographic breast density (%)				
Automated quantification	− 0.045	0.716	− 0.052	0.670
Manual quantification	− 0.330	< 0.01	− 0.273	< 0.05
Compressed breast thickness (mm)	0.648	< 0.01	0.467	< 0.01

DNPp-s, the difference of the minimal distance from the nipple to the pectoralis major muscle between the prone and supine MRI; DNIs-p, the difference of distance from the nipple to the internal thoracic artery between the prone and supine MRI

The manual quantification inversely correlates with both DNPp-s and DNIs-p, while the compressed breast thickness correlates with both DNPp-s and DNIs-p positively. *p* < 0.05 was considered as statistical significance

## Discussion

The present study demonstrated that automated and manual quantification of mammographic breast density acquired using mammodete^®^ was consistent with the 4-level classification recommended by QABCS. Among them, manual quantification showed an inverse correlation with the breast shape changes when the patient was prone versus supine in two directions on MRI. In addition, the compressed breast thickness during MMG correlated with the breast shape changes in the two directions with higher *r* values than the manual quantification of mammographic breast density. Therefore, the quantitative interpretation of MMG provided information about the biomechanical properties of the breast tissues, which were related to the degree of patient position-induced breast shape changes on prone and supine MRI.

The precise prediction of the breast shape changes is valuable for coinciding the MRI findings with the surgical findings, generating or simulating the supine MRI from the prone MRI, or predicting the breast deformity caused by some interventions, including neoadjuvant chemotherapy and breast-conserving therapy. Zolfagharnasab et al. [7] have regarded the breast density categories defined by the American College of Radiologists classification system (i.e., the Breast Imaging Reporting and Data System: BI-RADS) as the biomechanical properties of the breast that can correlate to the breast shape changes induced by breast-conserving surgery. In the present study, we used MMG to quantify the proportions of the fibroglandular tissue and fat within the breast and the compressed breast thickness, which might reflect the two biomechanical properties, the softness and size of the breast, respectively. Our study showed that the manual quantification of mammographic breast density and compressed breast thickness, which were acquired by MMG, correlated with breast shape changes. The merits of MMG are its low cost, high accessibility, wide range of view, and capability of retrospective analysis compared with MRI and ultrasonography, including their elastography techniques [14, 15].

Although both automated and manual quantification of mammographic breast density correlated with the 4-level classification recommended by QABCS, only the manual quantification provided the breast tissue softness related to the breast shape changes. The higher the value for manual quantification was, the less the breast shape changes, which suggests that the rich fibroglandular tissues can maintain the breast shape during the changes in the patients’ positions. MRI elastography and ultrasound elastography show that fat is softer than fibroglandular tissue [16]. Our findings suggest that manual quantification quantified the breast density accurately by excluding the effect of fat intervening within the fibroglandular tissue. The manual quantification provided quantitative information about the breast tissue softness, despite approximately 90% of the patients had scattered density or heterogeneously dense in the 4-level classification.

The compressed breast thickness, possibly reflecting the breast size, was the other MMG parameter that correlated with the breast shape changes. Gravity may significantly affect a large breast, leading to substantial shape changes when the patient is prone versus supine. In the present study, the *r* values of the compressed breast thickness were higher than those of the manual quantification of the mammographic breast density. Therefore, the simple MMG parameter is considered as a powerful indicator for predicting breast shape changes induced by the patients’ position rather than the quantification of mammographic breast density.

There were several limitations that should be addressed in this study. First, we excluded data from eight women in whom the supine MRI was done using the tilting table. Consequently, data from the patients with breast cancer in the outer portion of the breast were not included. Second, the T1-weighted imaging sequence differed between the prone and supine MRI. T1-weighted turbo spin-echo imaging used in the prone position is the standard technique owing to its high image quality. In the supine position, breath-hold T1-weighted 3D gradient-echo imaging with the identical spatial resolution to dynamic contrast-enhanced 3D imaging was utilized to confirm the coverage of this preoperative breast MRI. Then, the pixel size was approximately 0.22 mm^2^ smaller in the supine MRI than in the prone MRI. We did not assume that differences in the pixel size might have affected the results of this study because the values of differences were much smaller than DNP and DNI. Third, the changes in the breast shape in the craniocaudal direction were not analyzed because appropriate anatomical markers were difficult to find in this direction. Fourth, a single investigator analyzed the MMG using the dedicated MMG viewer. The conventional 4-level classification of MMG and manual quantification of the breast density might request the experience in MMG certificated by the QABCS. Fifth, the compressed breast thickness could be affected by the experience and skill of radiological technologists. Nevertheless, this simple parameter on MMG significantly correlated with the breast shape changes in the two directions on MRI. We assumed that the merits of the manual quantification of the mammographic breast density, which was also correlated to the breast shape changes, were its robustness to technologists’ skill and its relation to the biomechanical properties of the breast tissue in each patient. Sixth, we estimated the differences in DNP and DNI when the patient was prone and supine (i.e., DNPp-s and DNIs-p) but not the ratio of DNPp to DNPs, which has been evaluated as an indicator of the mobility of the breast by Satake et al. [17]. While the previous study shows some correlations between the tumor movement from the prone to supine positions and the ratio of DNPp to DNPs, the distance itself (i.e., DNPp-s and DNIs-p) should be estimated to extrapolate MRI findings for surgical procedures when the patient is supine. Last, we did not consider the effect of the size or location of breast cancer on the breast shape changes as done in a previous study [7]. Nonetheless, we excluded data from patients with breast cancers extending to the skin or pectoralis major muscle, where the effect on breast shape changes was obvious, and a patient with cancer ≥ 5 cm in diameter, which was too large to be an indication for breast-conserving surgery.

In conclusion, the breast shape changes associated with a change in patient position from prone to supine when measured by MRI are significantly correlated with compressed breast thickness on MMG. The manual quantification of the mammographic breast density, which may reflect the biomechanical properties of breast tissues, is also correlated with the breast shape changes. Therefore, quantitative interpretation of MMG is useful for predicting the degree of the patient position-induced breast shape changes in the two directions.
